# Supplementary figures and images for: Persistent advanced HIV disease in rural KwaZulu-Natal, South Africa: Trends, characteristics, and the urgent need for targeted interventions
Source: PLoS One. 2025 Feb 18;20(2):e0317674. doi: 10.1371/journal.pone.0317674 (PMC11835316; doi:10.1371/journal.pone.0317674)

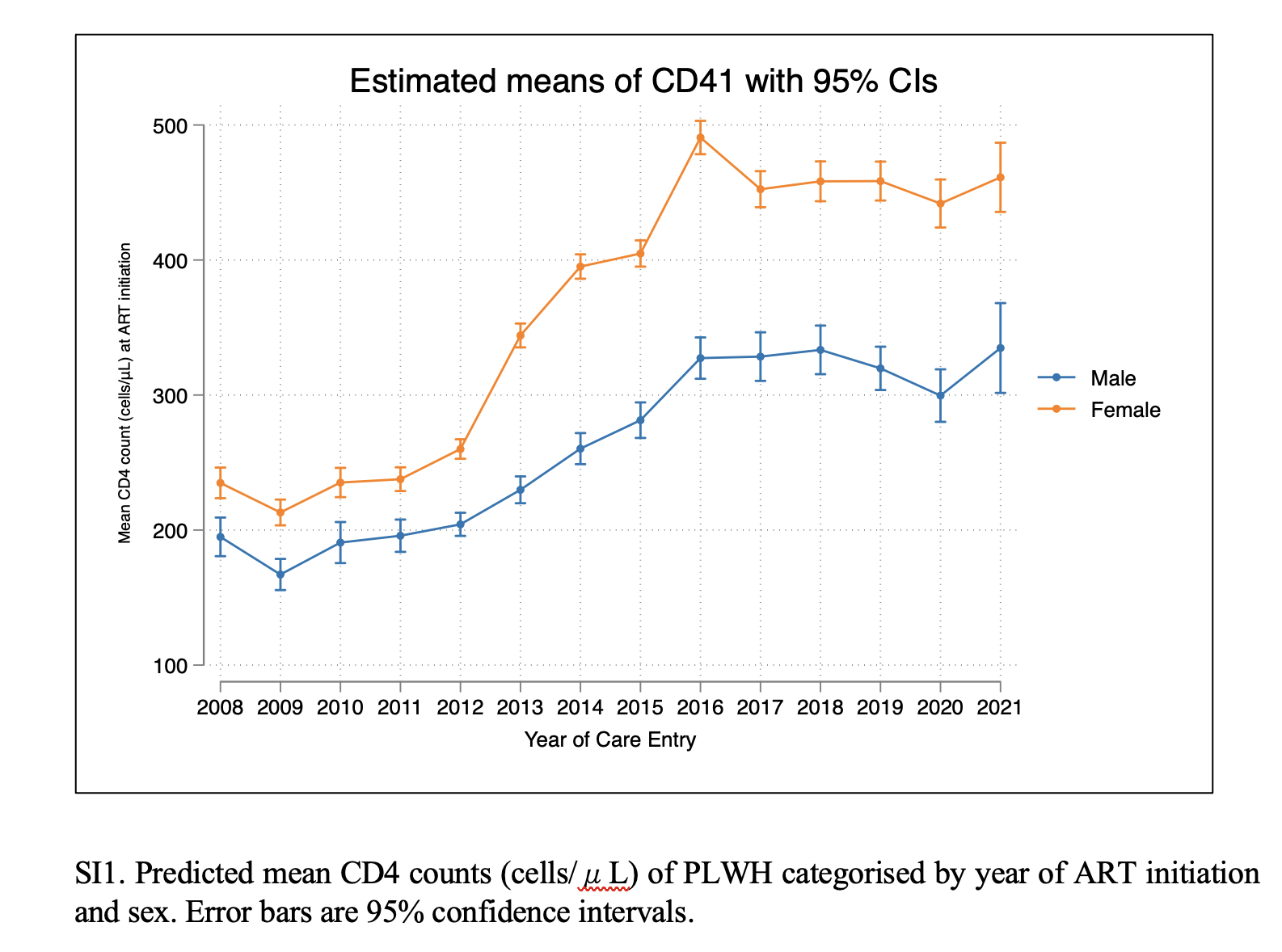

Supplement: S1 Fig — Error bars are 95% confidence intervals. (TIF) [file pone.0317674.s001.tif]

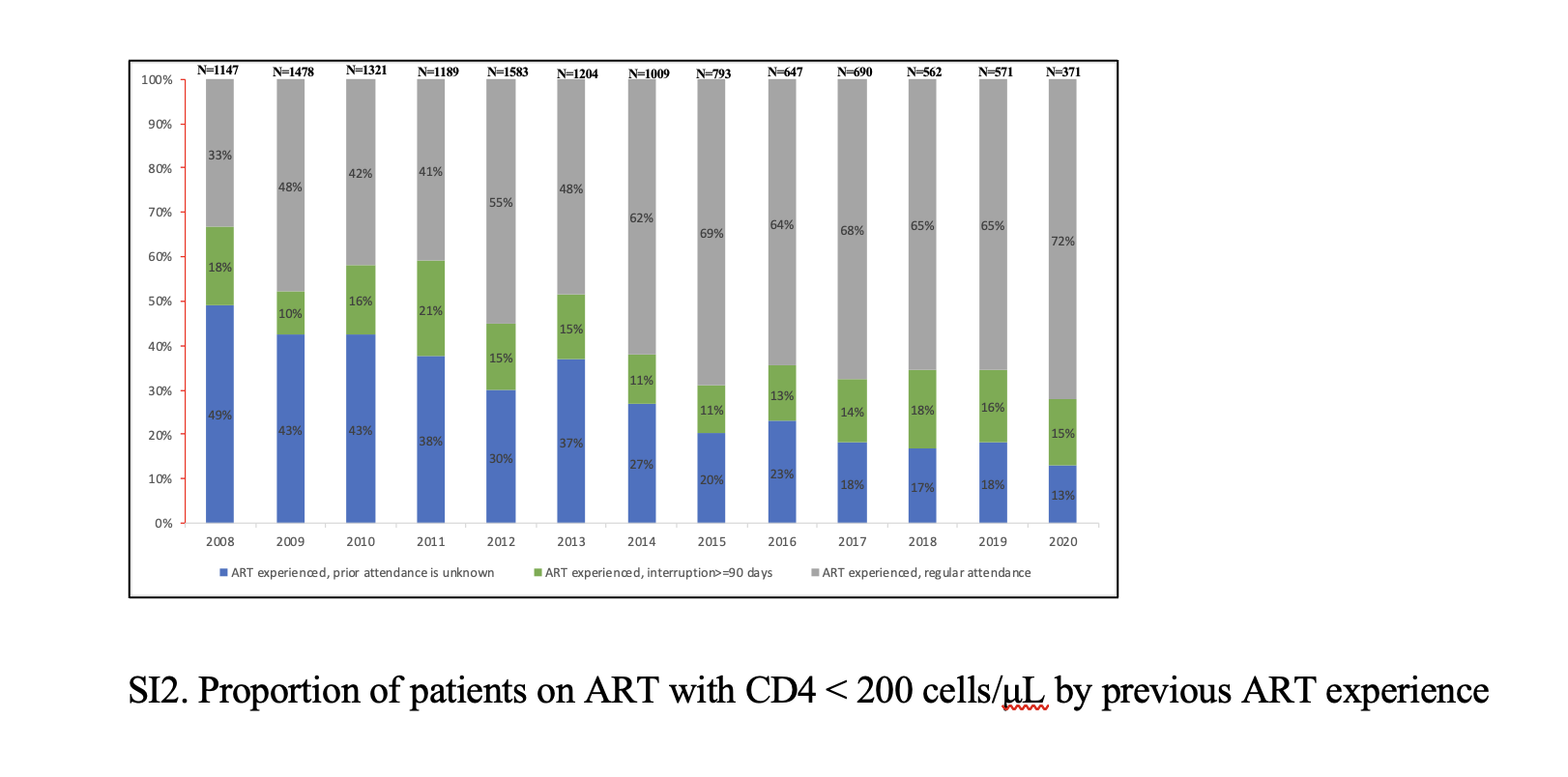

Supplement: S2 Fig — (TIF) [file pone.0317674.s002.tif]

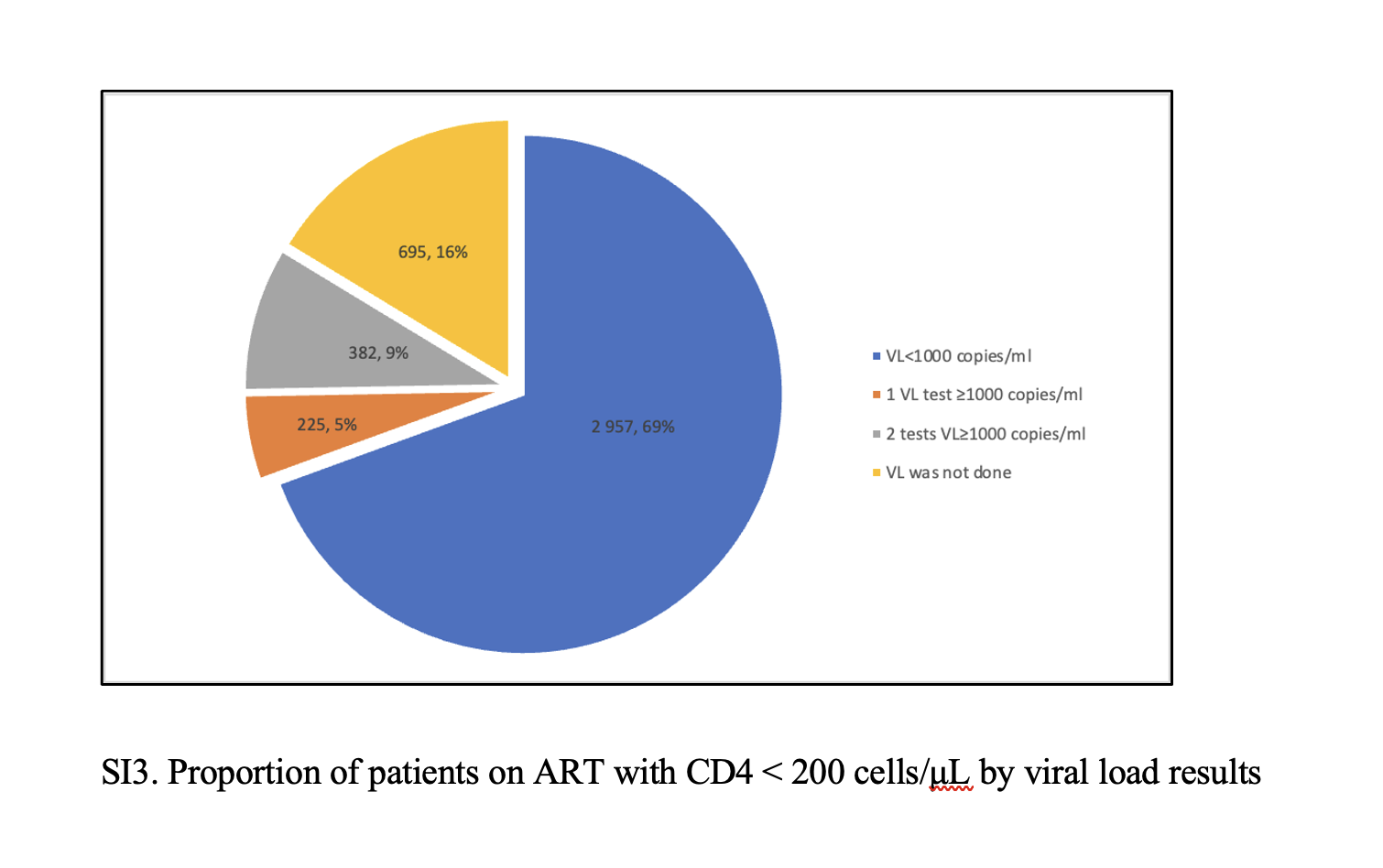

Supplement: S3 Fig — (TIF) [file pone.0317674.s003.tif]

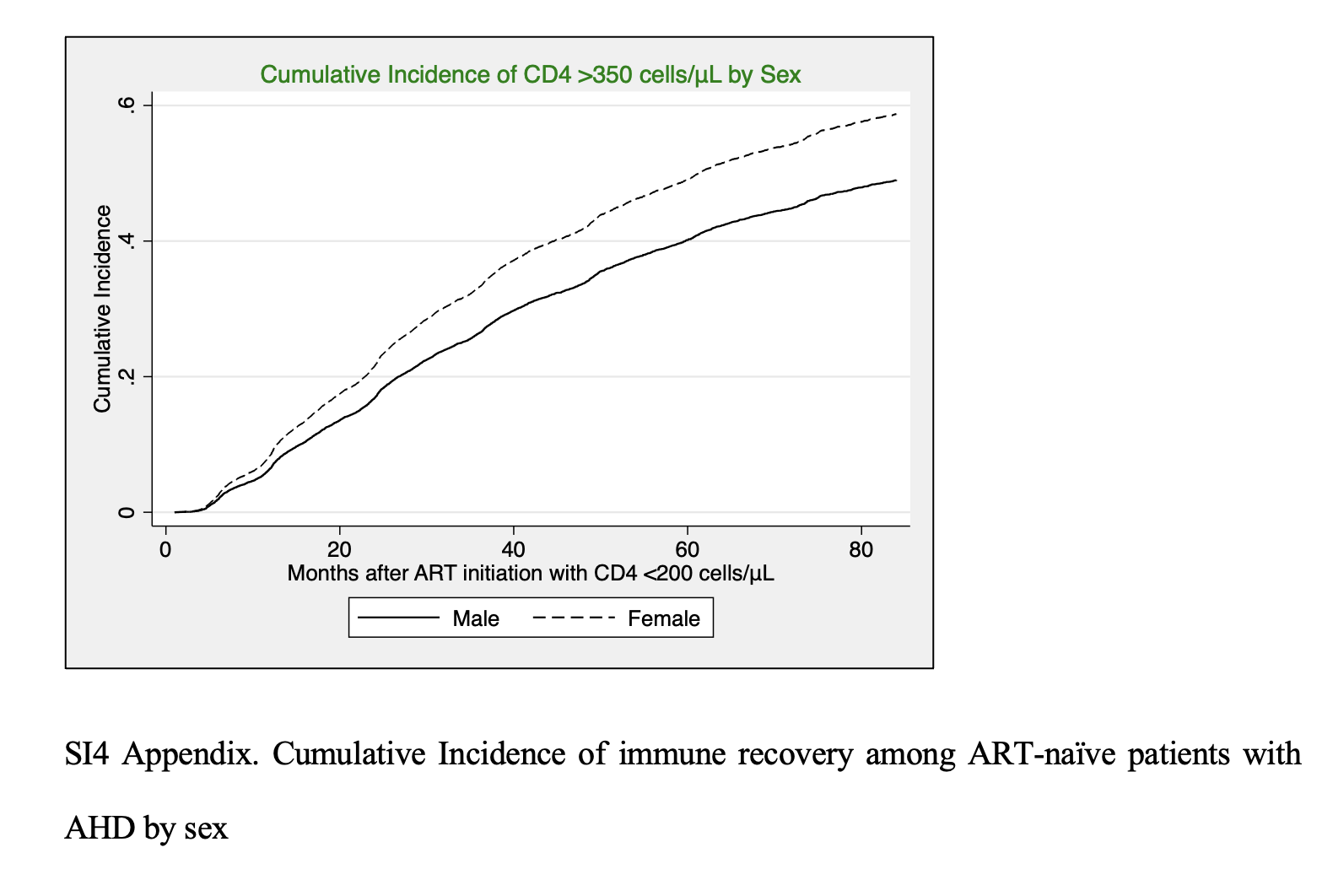

Supplement: S4 Fig — (TIF) [file pone.0317674.s004.tif]
