# Supplementary material for: Persistent advanced HIV disease in rural KwaZulu-Natal, South Africa: Trends, characteristics, and the urgent need for targeted interventions
Source: PLoS One. 2025 Feb 18;20(2):e0317674. doi: 10.1371/journal.pone.0317674 (PMC11835316; doi:10.1371/journal.pone.0317674)
Supplement: Attachment — Submitted filename: editor comment _kuwazulu.docx [file pone.0317674.s008.docx]

Persistent Advanced HIV Disease in Rural KwaZulu-Natal, South Africa: Trends,

Characteristics, and the Urgent Need for Targeted Interventions: PONE-D-24-02035R1

***General comments and suggestions***

Congratulation for the authors for trying to respond to the editor/peer reviewers’ comments and suggestions. It is well done. Yet, there are some remaining constructive suggestions or comments to deal with to further enrich the article. Please pay attention to reducing the size of the article through summarizing, merging and trimming sections; English language edits; and sticking to PLOSE ONE journal structure/requirement. [Submission Guidelines | PLOS ONE](https://journals.plos.org/plosone/s/submission-guidelines.#:~:text=The%20platform%20provides%20specialized%20tools%20for%20communicating%20technical%20details,%20including).

Also, the authors are anticipated to clearly describe and align the study design and analysis.

Otherwise, this is a n excellent finding of ADH in high HIV burden setting of South Africa that could be an input for province/national HIV policy*.*

***Specific comments and suggestions***

***KEY Words****: check if* Advanced HIV disease, AHD are to be retained-- both seem similar. Check if KwaZulu-Natal could be part of the key words.

**Abstract**

Check if PLWH is the right/standard abbreviation for persons living with HIV as compared to PLHIV

Check if the design, ‘’ A serial cross-sectional analysis’ is aligned with ‘’ FineGreys competing risks regression with proportional sub-distribution hazard models…’’ analysis. **PLEASE** review this issue again! Literally, this is a retrospective cohort study using the routine data (of ~13 years), as rightly mentioned under Ethical statement as,”…the study was retrospective in nature…”. Therefore, the burden of ADH could be described as both prevalence and incidence. The terms, ‘’trend, risk regression, hazard models, cumulative incidence, 100 adult-years follow-up time … ‘’ are usual used with cohort/longitudinal study designs.

Check if you need to add ’ medial’ before ‘’ … CD4 count was 277…’’.

Check if the statement, “Across all periods, those entering care with AHD were more likely to be men when compared to women” needs to be substantiated with statistically significant levels in the bracket.

Check punctuation and spacing in, ” In addition,,” and :” ARTexperienced” .

The authors should have line numbered all the document. No page numbers as well.

**Introduction section**

“Disease for those ART-naïve or returning to care after interruption in treatment) strongly predicts severe morbidity, comorbidities such as cryptococcal meningitis and Tuberculosis (TB), as well as mortality (11, 12). “

Check if this could be shortened to “Disease for those ART-naïve or returning to care after interruption in treatment strongly predicts severe morbidity, comorbidities and mortality (11, 12). “

**Methods section**

Check and correct the spacing in,” HIVinfected”, “ populationbased”, “ARTexperienced”. Please do same throughout the document.

The sentence,” KwaZulu-Natal, South Africa has 1.9 million PLWH, 1.1 million of whom are receiving ART (17).” is not clear. Check if it could be written as, “ KwaZulu-Natal province in South Africa has 1.9 million PLWH, 1.1 million of whom are receiving ART in xxxx (year/period) (17).’

**Result section** –well narrated yet needs to be condensed/summarized.

Please review this sentence, ”… and Having TB (AOR 2.46; 95% CI 2.19-2.76) was associated with higher odds of AHD at ART initiation (

Table 2).”

Check if heading write up fits to PLOS ONE criteria. E.g 3.1 Discussion, 3. Results ….

**Discussion section:-**The interpretation of the finding and the suggestion is well narrated.

Please restructure the discussion paragraph by paragraph (P).

Example: check if this structuring make sense--e.g P1:*17-24*, P2: *25-43* P3:*44-56* P4:*57-67* PY5:*67-73* PY6: *73-79* PY7: *80-88*

Also try to compare and contrast the findings with previous similar studies: For instance, **ART-experience and ADH,**

Make similar decimal point description of %. E.g line # 25-30: 36.6%, 62%, 43.45%. These used different decimal points. Suggested to write with same decimal point throughout.

**limitation:**

Refer to PLOS ONE journal requirements while narrating this section-which should be condensed/summarized/trimmed

Line # 100: you may replace ‘conclusion’ with “ interpretation”.

**Implications for practice and future research**

The content is very important. Yet need to be summarized and be part of discussion above. Again, please refer to PLOS ONE journal structure : [Submission Guidelines | PLOS ONE](https://journals.plos.org/plosone/s/submission-guidelines.#:~:text=The%20platform%20provides%20specialized%20tools%20for%20communicating%20technical%20details,%20including)
