# Supplementary material for: Persistent advanced HIV disease in rural KwaZulu-Natal, South Africa: Trends, characteristics, and the urgent need for targeted interventions
Source: PLoS One. 2025 Feb 18;20(2):e0317674. doi: 10.1371/journal.pone.0317674 (PMC11835316; doi:10.1371/journal.pone.0317674)
Supplement: Attachment — Submitted filename: PONE-D-24-02035.docx [file pone.0317674.s006.docx]

PONE-D-24-02035

Advanced HIV disease and CD4 in KZN 2008 – 2021

Ther authors report the prevalence and patterns of advanced HIV disease (AHD) at presentation to care between 2008 and 2021 in KwaZulu-Natal, South Africa. In addition, the cohort was followed-up to determine factors associated with CD4 recovery after entry into care with AHD. The proportion of people entering care with AHD decreases as the South African antiretroviral therapy (ART) policies become more inclusive. However, the numbers remain high and in later years, participants with AHD are more likely to be ART-experienced, especially men. CD4 recovery can occur in those who return to and remain in care for some time. This is an important topic as HIV services mature; its sampling of a rural population is a strength.

The paper is well-written but with several typos and missing words (lack of attention to detail). The formatting is all over the place. Line numbering only starts in the Discussion and there are no page numbers which has made the review more difficult.

The statistical tests are appropriate and the limitations are described.

General comments:

Please spell out the abbreviations at first use in the abstract, manuscript and tables.

Major comments.

The description of the study design as cross-sectional is a bit misleading as the cohort is followed-up and the analyses applied appropriately for longitudinal data. It would be more accurate to describe a cohort study and present the prevalence of AHD at entry into care (baseline) which is how the cohort is defined.

The description of table 1 is unclear and disorganized. Specify MEDIAN CD4 count. At one point MEAN is presented (S1) but MEDIAN has been used elsewhere. Where are the data on median/mean CD4 presented? In Table S1 why are the means presented when medians are reported in the text? I assume there is a difference between TIME ON ART and FOLLOW-UP TIME in the study. These are confused.

Throughout the analyses when age is used as a categorical variables and 18-24 years is the reference, only data for the 25–31 year category are presented when the effect on the OR/aOR is in the same direction for ALL age categories. it is not clear why 25-31 has been chosen. It could be presented as >24 years.

In all the Tables, please label how the data are presented in the table. E.g., n (%) or median (IQR) etc.

Table 3: I don’t understand the Males/Females with first CD4 test columns.

Line 1: is the CD4 recovery on the ART-naïve or ART-experienced group or both?

Line 6: TB at ART start or TB ever?

The Discussion is overly long and can be condensed. E.g., description of Universal Test and Treat policy; Implications and Future research para 2 repeats para 1

Minor comments

Background, third line: million is duplicated – both the figure and the word are used.

Background further down: ¾ - can’t be right; stage 3 or stage 4

Definitions and outcomes, 3^rd^ sentence: *and* who experienced a drop…

Statistical analysis, near the end: death and LTFU are competing *risks*, plural (not *a* competing risk, singular).

Results, 2^nd^ line: specify/describe the universal test and treat policy. Do not assume the reader is familiar with South African policy. The DATE/S need to be included.

Burden and trends of AHD among ART-experienced patients, line 4: the subject is missing from the sentence. What increased from 4% to 63%?

Before the Table 2 heading there is an incomplete sentence in bold. Why bold? Need to complete sentence.

Line 91: no subject. I think you mean selection *bias*

References:

Include the url and Access Date for on-line sources
