# Supplementary material for: Persistent advanced HIV disease in rural KwaZulu-Natal, South Africa: Trends, characteristics, and the urgent need for targeted interventions
Source: PLoS One. 2025 Feb 18;20(2):e0317674. doi: 10.1371/journal.pone.0317674 (PMC11835316; doi:10.1371/journal.pone.0317674)
Supplement: Attachment — Submitted filename: Rebuttal Letter-9 July 2024.docx [file pone.0317674.s007.docx]

9 July 2024

Dear reviewers,

Re: Responses to reviewers’ comments

Please find below a point-by-point response to the issues raised by the reviewers. The changes made to the manuscript are highlighted in track changes in the revised version.

Original Title: *Persistent Advanced HIV Disease in Rural KwaZulu-Natal, South Africa: Trend, Characteristics and Urgent Need for Targeted Interventions*.

# Reviewer 1

## General Comment

The authors report the prevalence and patterns of advanced HIV disease (AHD) at presentation to care between 2008 and 2021 in KwaZulu-Natal, South Africa. In addition, the cohort was followed up to determine factors associated with CD4 recovery after entry into care with AHD. The proportion of people entering care with AHD decreases as the South African antiretroviral therapy (ART) policies become more inclusive. However, the numbers remain high and in later years, participants with AHD are more likely to be ART-experienced, especially men. CD4 recovery can occur in those who return to and remain in care for some time. This is an important topic as HIV services mature; its sampling of a rural population is a strength.

The paper is well-written but with several typos and missing words (lack of attention to detail). The formatting is all over the place. Line numbering only starts in the Discussion and there are no page numbers which has made the review more difficult.

General comments:

Please spell out the abbreviations at first use in the abstract, manuscript and tables.

## Response

We express our gratitude to the reviewer for providing valuable feedback, and we acknowledge the validity of his comment. We sincerely apologize for the typos , missing words, and absence of line numbering. Consequently, we have diligently rectified this oversight.

Additionally, we have spelled out the abbreviations throughout the manuscript. For example see line 31 and 168.

## Major comments

The description of the study design as cross-sectional is a bit misleading as the cohort is followed up and the analyses applied appropriately for longitudinal data. It would be more accurate to describe a cohort study and present the prevalence of AHD at entry into care (baseline) which is how the cohort is defined.

## Response

We thank the reviewer for the comment. We omitted “serial” in line 30. Although we used longitudinal data, the study is a serial cross-sectional study as we aim to determine the prevalence of advanced HIV disease from 2008 to 2021, describe trends in advanced HIV disease in patients living with HIV across different time points, and investigate risk factors associated with advanced HIV disease in both ART-naïve and ART-experienced patients.

This has been adequately changed and now reads as follows in lines 30-31: “A serial cross-sectional analysis of annual CD4 count distribution was conducted among ART patients aged 18 years and older”

## Comment

The description of Table 1 is unclear and disorganized. Specify MEDIAN CD4 count. At one point MEAN is presented (S1) but MEDIAN has been used elsewhere. Where are the data on median/mean CD4 presented? In Table S1 why are the means presented when medians are reported in the text? I assume there is a difference between TIME ON ART and FOLLOW-UP TIME in the study. These are confused.

## Response

We thank the reviewer for the comment. As indicated in the statistical analysis “Continuous variables were summarised using mean and standard deviation if normally distributed, otherwise median, and interquartile range (IQR) were used…” a shown in line 153-154. For example, age was not normally distributed, so we used the median and interquartile range. However, for CD4, which was normally distributed, we used the mean. As such we have aligned the text with S1 Appendix and can be seen in lines 155-156, where it reads as follows: “The mean CD4 count increased across years, the largest increase in CD4 counts was observed in 2016…”

Additionally, as you suggested and to avoid confusion, we have changed “Follow-up on ART” to “Time on ART”. See Table 1.

## Comment

Throughout the analyses when age is used as a categorical variable and 18-24 years is the reference, only data for the 25–31 year category are presented when the effect on the OR/aOR is in the same direction for ALL age categories. it is not clear why 25-31 has been chosen. It could be presented as >24 years.

## Response

We thank the reviewer for the comment. We have made amendment as suggested. This is shown in line 195-199 and this reads as follows “*In the mixed-effect logistic regression, being male (Adjusted odds ratio[aOR] 1.80; 95% CI 1.71 to 1.90), PLWH in the age groups of 25-31 years, 32-38 years, and 39-45 years exhibited a higher odds of AHD at ART initiation when compared to those aged 18-24 years. The aOR for these age groups were 1.65 (95% CI 1.52 to 1.79), 2.15 (95% CI 1.98 to 2.34), and 2.22 (95% CI 2.03 to 2.44), respectively.”*

Additionally, we followed the guidelines provided by the International Journal of Epidemiology^[1]^ on forming categories, which state, “Age grouping should be mid-decade to mid-decade or five-year age groups.” This approach has been applied in this manuscript for the categorisation of age.

## Comment

In all the Tables, please label how the data are presented in the table. E.g., n (%) or median (IQR) etc.

## Response

We appreciate the reviewer's suggestion. We have added the missing labels to both Table 1 and Table 2.

## Comment

Table 3: I don’t understand the Males/Females with first CD4 test columns.

Line 1: is the CD4 recovery on the ART-naïve or ART-experienced group or both?

Line 6: TB at ART start or TB ever?

## Response

We appreciate the reviewer’s suggestion. We have adjusted Table 3 by separating the data for males, females, and the total. In table 4 we have expanded the title and this reads as follows “Table 1. Factors associated with CD4 recovery to >350 cells/μL following a CD4 <200 cells/μL from a competing risks regression model among ART-naïve and ART-experienced patients”. We have additionally expanded on this in lines 238-240 and it now reads as follows “Time to CD4 recovery to >350 cells/μL following a CD4 <200 cells/μL and outcomes post-identification of CD4 <200 cells/μL among ART-naïve and ART-experienced patients”.

## Comment

The Discussion is overly long and can be condensed. E.g., description of Universal Test and Treat policy; Implications and Future research para 2 repeats para 1

## Response

To shorten the discussion section, we have condensed and removed some repetitive paragraphs. See lines 298-302.

## Comment

Minor comments

Background, third line: million is duplicated – both the figure and the word are used.

## Response

We thank the reviewer for the comment. This has been adequately removed and It reads as follows “By 2021, it had reached 74% of all individuals infected with the human immunodeficiency virus (HIV), with 5,588,062 adults on ART”. See line 56-58.

## Comment

Background further down: ¾ - can’t be right; stage 3 or stage 4

## Response

We thank the reviewer for the comment. We have adequately addressed this and it reads as follows “Conversely, a CD4 count below 200 cells/μL or WHO stage 3 or 4 event (Advanced HIV Disease) strongly predicts severe morbidity, comorbidities such as cryptococcal meningitis and Tuberculosis (TB), as well as mortality”. See line 65-66.

## Comment

Definitions and outcomes, 3^rd^ sentence: *and* who experienced a drop…

Statistical analysis, near the end: death and LTFU are competing *risks*, plural (not *a* competing risk, singular).

## Response

We thanks the reviewer for the comment. As shown in lines 132-133, we have added the missing word, and it now reads as follows: “ART-experienced patients were those who entered care with CD4 count ≥ 200 cells/μL and who experienced a drop in CD4 count to <200 cells/μL during follow-up...”. Additionally, we have addressed misspelling of competing risks throughout the manuscript.

## Comment

Results, 2^nd^ line: specify/describe the universal test and treat policy. Do not assume the reader is familiar with South African policy. The DATE/S need to be included.

## Response

We appreciate the reviewer’s suggestion. We have removed the UTT. However, details on the Universal test and treat policy implementation in South Africa ins presented under ‘*study settings and participants’*. See line 97-101 and this reads as follows “*South Africa implemented the Universal Test and Treat (UTT) on September 1, 2016. Under this strategy, all public health facilities are required to scale up ART initiation for all HIV-infected individuals according to UTT guidelines, with an emphasis on providing same-day initiation for individuals newly diagnosed with HIV who are clinically and psychologically ready for lifelong ART(16)*”

## Comment

Burden and trends of AHD among ART-experienced patients, line 4: the subject is missing from the sentence. What increased from 4% to 63%.

## Response

We thank the reviewer for the comment. We have addressed this and it reads as follows “The proportion of patients with AHD who were ART-experienced increased from 4% in 2008 to 63% in 2021”. See line 41-42.

## Comment

Before the Table 2 heading there is an incomplete sentence in bold. Why bold? Need to complete sentence.

Response

We thank the reviewer for the comment. we have completed Table 2 heading and it reads as follows “Table 2. Factors associated with AHD among ART-naive patients at initiation of ART”. This also is reflected in the text as shown in line 200-201. Additionally, we have removed the bold typeface for all headings for tables and figures as suggested.

## Comment

Line 91: no subject. I think you mean selection *bias*

Response

We thank the reviewer for the comment. We have added the missing word as shown in line 375-381 and this reads as follows “This may have introduced selection bias and may limit the applicability of these findings to the entire population enrolled in the HIV program.”

# Reviewer 2

The study addresses an important public health issue in a setting with high HIV prevalence. It is well written and the team is commended for that. The following are my comments and suggestions

Comment
In the methods section, why did the authors choose Eshowe and Mbongolwane specifically, beyond HIV rates which are high in the whole of KwaZulu Natal?

## Response

We thank the reviewer for the comment. Eshowe is a small town that lies in the uMlalazi Municipality, King Cetshwayo district in KwaZulu Natal, South Africa.

We selected these areas primarily because of the high incidence and prevalence of HIV and TB compared to the rest of South Africa. The overall adult HIV prevalence in the area is 26.4% among adults aged 15-59 years. Additionally, between and 2019, Médecins Sans Frontières and the Department of Health implemented numerous community-based HIV/TB interventions. Following the implementation of the se interventions, a population-based survey conducted by MSF, these areas exceeded the UNAIDS 90-90-90 targets by achieving 90-94-95 in 2018. Despite this major achievement, programmatic data showed that the prevalence of AHD was still higher with 1 in 4 PLWH presenting to HIV care. Conclusions drawn from this study will be generalizable to other rural settings, especially those with high HIV prevalence and low-income.

Some of this information has been added under the study setting, as shown in lines 101-105 and 102-120, and it reads as follows: *“Since 2011, Médecins Sans Frontières (MSF) and the Department of Health (DoH) of KwaZulu-Natal have been implementing a community-based HIV/TB project. The project involves 10 primary health facilities and two hospitals in the Eshowe and Mbongolwane areas. The areas are predominantly rural areas with one semi-urban market town, with a combined population of 114,490 in 2021 (18). According to a population-based survey conducted by MSF, these areas exceeded the UNAIDS 90-90-90 targets by achieving 90-94-95 in 2018. The survey also revealed that HIV prevalence in the study areas was an estimated 26.4 among 15-59 years old adults (15)”.*

It is against this background, we conducted this study solely aiming at determining the burden of and treatment outcomes of advanced HIV disease among patients starting ART and those ART-experienced in the Eshowe and Mbongolwane areas.

Comment
2. Justify the use of and assumptions for the mixed effect regression and Poisson models

## Response

We thank the reviewer for the comment. The primary reason we used mixed effect logistic regression is that we assumed a random effect for each facility. Given that the study was conducted at 10 public health facilities, we used mixed effect logistic regression to model binary outcomes variables (assessing the probability of having AHD among ART-naïve patients at ART initiation) , in which the odds of the outcomes are modelled as linear combination of the predictor variables when data from these facilities are clustered. Additionally, given some CD4 data were missing. Mixed-effect logistic regression models are mostly based on the assumption that missing data are random and non-informative.

While, Poisson regression model allows to assess epidemiological studies when the main outcome is an integer. It assumes that these types of outcomes are random variables with positive integers and are distributed as the *Poisson probability distribution^[2]^*. The Poisson regression model is used in longitudinal cohort to estimate the expected value of outcomes among exposure and non-exposure groups, adjusted for the effect of potential confounders. One advantage of this model is that it can be used to obtain an estimate of the relative risk (RR), adjusted for potential confounders. Based on the aforementioned reasons, we opted to utilize both mixed-effect logistic regression models and Poisson regression models. The former was employed to determine the odds of AHD among ART-naïve patients at ART initiation, adjusted for age, sex, calendar year, and TB status. Meanwhile, the latter was utilized to identify predictors of AHD among ART-experienced patients, with relative risk serving as the measure of effect.
Comment
The last sentence here is incomplete: Factors associated with AHD among ART-naïve patients.
In the mixed-effect logistic regression, being male (aOR 1.80; 95% CI 1.71 to 1.90), PLWH
aged 25-31 years (aOR1.65; 95% CI 1.52-1.79) and having TB (AOR 2.46; 95% CI 2.19-2.76)
had higher odds of AHD at ART start.

## Response

We appreciate the reviewer’s comment. We have completed the sentence as shown in line 195-201 and it now rears as follows *“In the mixed-effect logistic regression, being male (Adjusted odds ratio[aOR] 1.80; 95% CI 1.71 to 1.90), PLWH in the age groups of 25-31 years, 32-38 years, and 39-45 years exhibited a higher odds of AHD at ART initiation when compared to those aged 18-24 years . The aOR for these age groups were 1.65 (95% CI 1.52 to 1.79), 2.15 (95% CI 1.98 to 2.34), and 2.22 (95% CI 2.03 to 2.44), respectively. PLWH aged 25-31 years (aOR1.65; 95% CI 1.52-1.79) and Having TB (AOR 2.46; 95% CI 2.19-2.76) was associated with higher odds of AHD at ART initiation (Table 2).”*

Comment
The pages are not numbered

## Response

We thank the reviewer for the comment and apologize for this omission. We have added the page number and line numbers.

Comment
Resolve formatting issues on multiple pages that are distracting when reading an otherwise well written manuscript

## Response

We appreciate the reviewer for the comment and apologize for the formatting issues. We have resolved all formatting issues at the best of our ability.

## Comment

The statement: "Finally, having started ART between 2012 and 2015 (aSHR 0.60; 95% CI 0.56 to 0.65) or between 2016 and 2021 (aSHR 0.54; 95% CI 0.49-0.59) compared to having started ART between 2008 and 2011" is incomplete.

## Response

We appreciate the reviewer’s comment. We have completed the sentence as shown in line 258-261 and it now rears as follows *“Starting ART between 2012 and 2015 (aSHR 0.60; 95% CI 0.56 to 0.65) or between 2016 and 2021 (aSHR 0.54; 95% CI 0.49 to 0.59) compared to starting ART between 2008 and 2011 was associated with a reduced relative incidence of CD4 recovery to >350 cells/μL.”*

Comment

It would be useful to provide a statistic in place of the words "consistently high" in the opening statement of the discussion.

## Response

We thank the reviewer for their comment. We have made the suggested adjustment, and the revised text now reads as follows as shown in line 267-270 “*Our findings indicate that although the proportion of PLWH entering care with AHD in rural KwaZulu-Natal, South Africa, has decreased over 12 years, it has remained consistently high (affecting 1 in 4 PLWH presenting to HIV services) despite the expansion of HIV testing services and the successful implementation of ART scale-up.*”

## Comment

In your discussion of implications for the study, you mention only differentiated care models, but other options are available and may be worth mentioning, such as community support programs, digital adherence tools and so on. There is a need to broaden the options to solve this problem.

## Response

We thank the reviewer for their comment. We have made the suggested adjustment, and the revised text now reads as follows as shown in line 425-444 “*Addressing AHD among ART-experienced patients will require improving and maintaining consistent long-term adherence to ART and viral suppression through various interventions such as text messaging, counselling, and mobile phone technologies. A network meta-analysis evaluating the comparative effectiveness of adherence interventions to increase adherence to ART found that text messaging, counselling, support, and the additive effects of behavioural and cognitive interventions were associated with increased adherence to ART, relative to standard care (55). However, the adoption and adaptation of these interventions according to resource availability and program outcomes in routine practice remain to be seen. As ART rollout expands in LMICs, health systems need to continually adjust to accommodate further expansion. Data suggest that community versus facility-based models of ART delivery, coupled with the concurrent implementation of several differentiated models of HIV care in large ART programs, result in at least comparable outcomes (ART adherence, retention in HIV care, and viral suppression) for clinically stable PLWH on treatment in LMICs. These models are likely to be cost-effective and feasible (56, 57)*.”

# Reviewer 3

## Comment

Background section
Paragraph 1
• Reference 1 does not contain the quoted stats. It might be better to use the WHO country data: <https://www.who.int/data/gho/data/indicators/indicator-details/GHO/reported-number-of-people-receiving-antiretroviral-therapy>
5 588 062 people in SA on ART in 2021
o <https://www.who.int/data/gho/data/indicators/indicator-details/GHO/estimated-antiretroviral-therapy-coverage-among-people-living-with-hiv>-(-)
75% ART coverage in SA in 2022
• The “Treat all policy” was known as the “Universal Test and Treat policy” in South Africa (2016); it is available at:
<http://www.kznhealth.gov.za/test_treat.htm>
<https://sahivsoc.org/Files/PREP%20and%20TT%20Policy%20-%20Final%20Draft%20-%205%20May%202016%20>(HIV%20news).pdf
<https://sahivsoc.org/Files/22%208%2016%20Circular%20UTT%20%20%20Decongestion%20CCMT%20Directorate.pdf>
These might be better references for implementation of UTT policy in SA.

## Response

We thanks the suggestion and provision of the crucial links. We have inserted the WHO country profile as suggested and this shown in line 57-58. For refence pertaining to the Universal test and treat , we have included refence to the implementation of Universal test and Treat policy in South Africa and this is shown in line 97-101.

## Comment

Background, Paragraph 2
• WHO ¾ stage should be re-written as WHO stage 3 or 4

## Response

We thank the reviewer for the comment. We have adequately addressed this and it reads as follows “Conversely, a CD4 count below 200 cells/μL or WHO stage 3 or 4 event (Advanced HIV Disease) strongly predicts severe morbidity, comorbidities such as cryptococcal meningitis and Tuberculosis (TB), as well as mortality”. See line 65-68.

## Comment

Setting and participants section
• “…of which only 1.1 million are receiving…”, should be re-phrased as “…1.1 million of whom are receiving…”

## Response

We appreciate the reviewer’s suggestion. We included this in the manuscript as shown inline 101-102 and this reads as follows “*KwaZulu-Natal, South Africa has 1.9 million PLWH, 1.1 millions of whom are receiving are receiving ART”*

## Comment

• Eshowe and Mbongolwane are mentioned under study setting, but not rationale is given as to why these populations were chosen for the study. Especially if you are drawing conclusion on AHD in rural areas, an attempt should be made to describe the rurality of the setting, and the generalizability to other rural settings.
• No context is given on the relationship between umlalazi municipality (mentioned in the Data Sources section) and Eshowe and Mbongolwane.

## Response

We thank the reviewer for this comment. This study was conducted in the rural areas of Eshowe and Mbongolwane in KwaZulu-Natal, South Africa. We selected these areas primarily because of the high incidence and prevalence of HIV and TB compared to the rest of South Africa. The overall adult HIV prevalence in the area is 26.4% among adults aged 15-59 years. Additionally, between and 2019, Médecins Sans Frontières and the Department of Health implemented numerous community-based HIV/TB interventions. Following the implementation of the se interventions, a population-based survey conducted by MSF, these areas exceeded the UNAIDS 90-90-90 targets by achieving 90-94-95 in 2018.Therefore, the conclusions drawn from this study will be generalizable to other rural settings, especially those with high HIV prevalence and low-income. Some of this information has been added under the study setting, as shown in lines 101-105 and 102-117, and it reads as follows: *“Since 2011, Médecins Sans Frontières (MSF) and the Department of Health (DoH) of KwaZulu-Natal have been implementing a community-based HIV/TB project. The project involves 10 primary health facilities and two hospitals in the Eshowe and Mbongolwane areas. The areas are predominantly rural areas with one semi-urban market town, with a combined population of 114,490 in 2021 (18). According to a population-based survey conducted by MSF, these areas exceeded the UNAIDS 90-90-90 targets by achieving 90-94-95 in 2018. The survey also revealed that HIV prevalence in the study areas was an estimated 26.4 among 15-59 years old adults (15)”.*

Furthermore, to avoid confusion, we have removed information related to Umlalazi Municipality, as shown in line 125.

## Comment

Statistical analysis Section
• The use of the Fine-Gray competing risks regression with a proportional sub-distribution hazard (SHR) model should perhaps be references as some readers may be unfamiliar with the method.

## Response

We thank the reviewer for this comment. We have added references as recommended. And this shown in line 169 and 170 and it reads as follows *“…and assess the overall impact of covariates on the incidence of CD4 recovery to >350 cells/μL, treating death and LTFU as a competing risk (21, 24).”*

## Comment

Results section, paragraph In the multivariable competing risk regression model of time to CD4 recovery >350 cells/μL following a CD4 <200 cells/μL adjusted for clustering at the health facility, lines 1 to 10.

“These factors were associated with a reduced risk of CD4 recovery to >350 cells/μL.” It is unclear what factors you are referring to, as all above-mentioned factors has a better CD4 > 350 recovery.

## Response

We appreciate the reviewer for the comment. We have rephrase the sentence as shown in line 258-261 and this now reads as follows “*Starting ART between 2012 and 2015 (aSHR 0.60; 95% CI 0.56 to 0.65) or between 2016 and 2021 (aSHR 0.54; 95% CI 0.49 to 0.59) compared to starting ART between 2008 and 2011 was associated with a reduced relative incidence of CD4 recovery to >350 cells/μL*”

## Comment

There was also a better CD4 > 350 recovery on non-stavudine containing regimens, which is not mentioned here.

## Response

We appreciate the reviver’s comment. We have paraphrased the interpretation of results relative to this specific outcome as shown in line 262 and 265 and this reads as follows “*Finally, patients who were taking ABC+3TC (aSHR 0.51; 95% CI 0.30 to 0.88) , TDF+FTC/3TC (aSHR 0.75; 95% CI 0.30 to 0.88) and AZT+3TC (aSHR 0.92; 95% CI 0.85 to 0.98) containing regimen were associated with a relative incidence of CD4 recovery to >350 cells/μL compared to Stavudine+3TC/FTC).* ”

## Comment

Table 1:
• “Stavudine” is misspelled
• “ART start” should be changed to “ART initiation” for consistency of terminology.

## Response

We thank the reviewer for the comment. We have corrected the misspelling and changed 'ART start' to 'ART initiation' in Table 1. Additionally, Stavudine has been corrected throughout the manuscript.

**Comment**

Discussion Section, line 12 to 19
• The description of AHD in the rural setting, should be contrasted with the existing literature about urban settings.

## Response

We thank the reviewer for the comment. Line 267-275 covers summary of the key findings of the manuscript and we believe this should remain unchanged, given there is a paragraph contrasting the higher prevalence of AHD in other rural setting. This is shown in line 278-279 and it reads as follows “*Notably, certain studies in SSA indicate even higher proportions of AHD at diagnosis, reaching up to 62% in rural Tanzania and Ethiopia (29-31)*”

Comment
Discussion Section, line 30
• “September 2015” should be corrected to “2016”.
The SA DoH guideline of 2015 set the CD4 threshold at 500, available here:
<https://sahivsoc.org/Files/ART%20Guidelines%2015052015.pdf>

## Response

We appreciate the reviewer’s comment. We have corrected the year of the UTT implementation of UTT as shown line 294-297 and this now reads as follows “*However, eligibility expanded to higher CD4 thresholds, and in September 2016, a "treat-all policy" guidance was introduced, recommending immediate treatment for all individuals upon HIV diagnosis.*”

## Comment

The other factors besides gender, which affect CD4 recovery i.e. age group, ART regimen, time period in which ART was initiated, being on TB treatment – also warrant further discussion.

## Response

We thank the reviewer for the comment, we have discussed about these factors as shown in line 357-361 and this reads as follows *“Our data also suggest that CD4 recovery to >350 cells/μL was higher in those on TB treatment, consistent with previous evidence (55) showing that PLWH presenting with TB experience immune recovery after starting ART that is no worse than in other patient groups studied in South Africa. In contrast, we observed slower CD4 recovery to >350 cells/μL in younger individuals (51)”*.

Comment
Figure 2
Suggestion: Add overlying labels on the figure, showing the changes in national policy on CD4 threshold at ART initiation. The decline in AHD does follow the policy changes from CD4 of 200 in 2008, to 350 in 2013, to 500 in 2014, then to UTT in 2016.
Sahivsoc.org has the old DOH policies, if you are looking for them.

## Response

Thank you for your suggestion. We have added the overlying labels on the figure as suggested.

Comment
S1 Appendix.
• The title: Predicted average relative risk of Males with First CD4 count <200 cells/mL
by age group. Does not match the info on the graph.
The y-axis on the graph should specify whether that is the CD4 at initiation or whether it is the change in CD4.

## Response

We appreciate the reviewer for the comment , we have made the necessary changes and this reads as follows “Predicted mean CD4 counts (cells/μL) of PLWH categorized by year of ART initiation and sex”.

## Comment

S2 appendix and S3 appendix
• It is unclear what is being demonstrated here. Perhaps these percentages should be accompanied by absolute numbers. Or another analysis should be done - % of people on ART with no interruption, and VL <1000 with a CD4<200, over time.
This will tell you whether there is a truly increasing burden of failure of immune recovery while on effective ART, (which I highly doubt there was)
The data is the current format don’t give that information, as there was an increasing number of people on ART over time. So the proportion in that category will logically increase over time.

## Response

Thank you for your suggestion. We have included absolute numbers in the S2 appendix. S3 appendix , absolute numbers are provided under data labels in the.

# Reviewer 4

## Comment

Thank you for the opportunity to review this manuscript on advanced HIV disease in a rural population in KZN. The manuscript describes an important topic around which renewed focus is needed, and these are valuable data to add to the evidence body.
The presentation and readability of the results section of the manuscript could be improved.

## Response

We express our gratitude to the reviewer for providing valuable feedback, and we acknowledge the validity of their comment.
Comment
Background
Amend “WHO ¾ stage” to read “WHO stage 3 / 4” or “3 or 4”

## Response

We thank the reviewer for the comment. We have adequately addressed this and it reads as follows “Conversely, a CD4 count below 200 cells/μL or WHO stage 3 or 4 event (Advanced HIV Disease) strongly predicts severe morbidity, comorbidities such as cryptococcal meningitis and Tuberculosis (TB), as well as mortality”. See line 65-68.

## Comment

Methods
Your inclusion criteria state “Our study included all individuals
aged 18 years and **older** living with HIV who were receiving ART, and who had undergone
baseline and subsequent CD4 cell measurements between January 2008 and June 2021.” Please define what baseline is in this case.

## Response

We appreciate the reviewer for the comment. We have rephrased this and it reads as follows “*Our study included all individuals aged 18 years and older living with HIV who were receiving ART and had undergone a baseline CD4 measurement when entering HIV care, along with subsequent CD4 cell measurements between January 2008 and June 2021.”* . See line 117-120.

Comment
Although it is mentioned in the limitations of the study, it would be useful to include in the results (in Figure 1) the numbers of adults on ART who were excluded because they did not have a CD4 cell count at ART initiation and the numbers without a subsequent CD4 measure. With the introduction of treat all CD4 testing has declined, and this could introduce bias into the results, since those who receive CD4 testing are sicker. It would help the reader assess the potential for bias in your results.

## Response

We thank the reviewer for the comment. We have added a box in the figure showing the number of individuals excluded due to missing CD4 count results at ART initiation. As you mentioned, we have thoroughly discussed the implications of declining CD4 testing due to changes in guidelines.

## Comment

Last sentence under ‘Data sources and variable’ is missing a word.
Response

We thank the reviewer for the comment. We added the missing word and this reads as follows as shown line 127-128 “*We utilised demographic and clinical data documented at ART initiation as well as during follow-up.”.*

Comment
Results
The title for Figure S1 is incorrect

## Response

We appreciate the reviewer for the comment , we have made the necessary changes and this reads as follows “Predicted mean CD4 counts (cells/μL) of PLWH categorized by year of ART initiation and sex”.

Comment
Table 3 may be better presented as a graph

**Response**

We appreciate the reviewer’s suggestion. While we acknowledge there is an excess of information in Table 3, we wanted to compare different CD4 categories between females and males, as well as assess the relative risk of males entering care with a CD4 count of < 200 cells/μL compared to females during the study period. The aforementioned information cannot be adequately and meaningfully presented in a graph.

Comment
Second last sentence of results is incomplete: “Finally, having started ART between 2012 and 2015 (aSHR 0.60; 95% CI 0.56 to 0.65) or between 2016 and 2021 (aSHR 0.54; 95% CI 0.49-0.59) compared to having started ART between 2008 and 2011.”

## Response

We thank the reviewer for the comment. We have adequately addressed as shwon in line 258-261 and it reads as follows “*Starting ART between 2012 and 2015 (aSHR 0.60; 95% CI 0.56 to 0.65) or between 2016 and 2021 (aSHR 0.54; 95% CI 0.49 to 0.59) compared to starting ART between 2008 and 2011 was associated with a reduced relative incidence of CD4 recovery to >350 cells/μL.”*

## Comment

It may be better to summarize the factors associated with reduced risk of CD4 recovery without providing the aSHRs in the text, to improve readability.

## Response

We have rephrased the sentence as follows (as noted in our response). Lines 262-265 now read as follows “*Finally, patients who were taking ABC+3TC (aSHR 0.51; 95% CI 0.30 to 0.88) , TDF+FTC/3TC (aSHR 0.75; 95% CI 0.30 to 0.88) and AZT+3TC (aSHR 0.92; 95% CI 0.85 to 0.98) containing regimen were associated with a relative incidence of CD4 recovery to >350 cells/μL compared to Stavudine+3TC/FTC).* ”

Comment
Please clarify what prior attendance unknown means?
Response

We thank the reviewer for the comment. The S2 appendix examines the proportion of ART-experienced patients with AHD by their previous ART experience. We categorized the patients into those who had an interruption in care of ≥90 days and those with regular attendance. However, one group had no documented attendance (neither ≥90 days interruption in care nor regular attendance). Reason why we labelled these patients as prior attendance unknown. We have added this under ‘*definition and outcomes*’ as shown in line 138-140 and it reads as follows “*We also defined prior attendance unknown if the patients had neither a ≥90-day interruption in care nor regular attendance or continuously in care.*”

## Comment

Results show later year of ART start reduced risk of AHD on ART, could this be as a result of less follow-up time? Would be worth adding to the discussion about this result

**Response**
We appreciate the reviewer's comment and acknowledge your suggestion to expand the discussion, especially regarding relationship between the year of ART initiation and reduced risk of AHD among ART-experienced patients. However, feedback from other reviewers suggested shortening the discussion as it was too lengthy. For this reason, we could not expand the discussion to include an analysis of factors associated with the year of ART initiation.

One possible explanation is that many patients who began ART in later years did so with higher CD4 counts due to changes in guidelines, as comprehensively discussed in detail in the section (refer to lines 291-297).

**Comment**

Discussion
More discussion about the ART-experienced patients with AHD would be beneficial. From your Figure S2 it looks like an increasing proportion are in care (ART experienced, regular attendance) and not treatment interrupters, and the majority (69%) were virally suppressed. Some further clarity on these results is needed in the discussion.

## Response

We thank the reviewer for the comment. We have added a section pertaining to the issues raised , this is shown in line 335-341 and it reads as follows “*Additionally, among those ART-experienced patients with AHD with documented VL, 69% were virally suppressed. This demonstrates that in a high HIV-prevalence setting such as South Africa, a significant number of PLWH with AHD may not be identified if viral load testing is used alone. This finding contributes to the growing literature on patients who experience immunosuppression despite ART and virologic response. The subset of PLWH experiencing AHD despite prolonged ART and viral load suppression has been noted in several other studies from the region.*”

1. Reijneveld S. Age in epidemiological analysis. BMJ Publishing Group Ltd; 2003. p. 397-.
2. Suárez EL, Pérez CM, Nogueras GM, Moreno-Gorrín C. Biostatistics in public health using STATA: CRC Press; 2016.
