# Supplementary material for: Persistent advanced HIV disease in rural KwaZulu-Natal, South Africa: Trends, characteristics, and the urgent need for targeted interventions
Source: PLoS One. 2025 Feb 18;20(2):e0317674. doi: 10.1371/journal.pone.0317674 (PMC11835316; doi:10.1371/journal.pone.0317674)
Supplement: Attachment — Submitted filename: Rebuttal Letter-15 October 2024.docx [file pone.0317674.s010.docx]

27 October 2024

Dear reviewers,

Re: Responses to reviewers’ comments

Please find below a point-by-point response to the issues raised by the reviewers. The changes made to the manuscript are highlighted in track changes in the revised version.

Original Title: Persistent Advanced HIV Disease in Rural KwaZulu-Natal, South Africa: Trend, Characteristics and Urgent Need for Targeted Interventions.

**General Comment**

***General comments and suggestions***

Congratulation for the authors for trying to respond to the editor/peer reviewers’ comments and suggestions. It is well done. Yet, there are some remaining constructive suggestions or comments to deal with to further enrich the article. Please pay attention to reducing the size of the article through summarizing, merging and trimming sections; English language edits; and sticking to PLOSE ONE journal structure/requirement. [Submission Guidelines | PLOS ONE](https://journals.plos.org/plosone/s/submission-guidelines.#:~:text=The%20platform%20provides%20specialized%20tools%20for%20communicating%20technical%20details,%20including).

Also, the authors are anticipated to clearly describe and align the study design and analysis.

Otherwise, this is a n excellent finding of ADH in high HIV burden setting of South Africa that could be an input for province/national HIV policy*.*

**Response**

We express our gratitude to the reviewer for their valuable feedback and acknowledge the validity of the comment. We have reduced the length of the manuscript by summarizing, merging, and trimming down certain sections throughout the manuscript.

**Comment 1**

***Specific comments and suggestions***

***KEY Words****: check if* Advanced HIV disease, AHD are to be retained-- both seem similar. Check if KwaZulu-Natal could be part of the key words.

**Response**

We appreciate the reviewer's suggestion. We have deleted AHD and added KwaZulu-Natal part of the keywords, and this is shown in line 20-21 and it reads as follows “*keywords: Disease burden, Advanced HIV disease, ART-naïve, ART-experienced, KwaZulu-Natal, South Africa.*”

**Comment 2**

**Abstract**

Check if PLWH is the right/standard abbreviation for persons living with HIV as compared to PLHIV

**Response**

Thank you to the reviewer for the comment. We have replaced "PLWH" with "PLHIV" throughout the manuscript.

**Comment 3**

A serial cross-sectional analysis’ is aligned with” FineGreys competing risks regression with proportional sub-distribution hazard models…’’ analysis. PLEASE review this issue again! Literally, this is a retrospective cohort study using the routine data (of ~13 years), as rightly mentioned under Ethical statement as,”…the study was retrospective in nature…”. Therefore, the burden of ADH could be described as both prevalence and incidence. The terms, ‘’trend, risk regression, hazard models, cumulative incidence, 100 adult-years follow-up time … ‘’ are usual used with cohort/longitudinal study designs.

**Response**

Thank you to the reviewer for the suggestion. We have revised this to "retrospective cohort," as reflected in lines 31 and 93, which now read as follows: “*This was a retrospective cohort design of annual CD4 cell count data among adults receiving ART between January 1, 2008, and June 30, 2021, using routinely collected ART program data at 10 public health facilities.”*

**Comment 4**

Check if you need to add ’ medial’ before ‘’ … CD4 count was 277…’’.

**Response**

We thank the reviewer for the suggestion. We have added median as shown in line 171-173 and it reads as follows “*A total of 34,729 patients were included of which 68.5% were females. The median age of the study sample was 33.5 years (interquartile range [IQR] 27-41 years), and the median CD4 count was 277 cells/μL (IQR, 149-452 cells/μL).*”

**Comment 5**

Check if the statement, “Across all periods, those entering care with AHD were more likely to be men when compared to women” needs to be substantiated with statistically significant levels in the bracket.

Check punctuation and spacing in,” In addition,” and:” ART-experienced”.

**Response**

Thank you for the reviewer’s insightful comment. We have added supporting statistical information to strengthen the statement, as detailed in line 42-43 and it reads as follows *“Across all periods, those entering care with AHD were more likely to be men when compared to women (Relative risk [RR] 1.49 ; 95% 1.33 to 1.67). In addition, the proportion of patients with AHD who were ART-experienced increased from 4% in 2008 to 63% in 2021.”*

**Comment 6**

The authors should have line numbered all the document. No page numbers as well.

**Response**

We appreciate the reviewer’s comment, we have expanded the line number to all pages and have added number

**Comment 7**

**Introduction section**

“Disease for those ART-naïve or returning to care after interruption in treatment) strongly predicts severe morbidity, comorbidities such as cryptococcal meningitis and Tuberculosis (TB), as well as mortality (11, 12). “

Check if this could be shortened to “Disease for those ART-naïve or returning to care after interruption in treatment strongly predicts severe morbidity, comorbidities and mortality (11, 12). “

**Response**

We thank the reviewer for the suggestion. The sentence has been revised as per the recommendation on line 70, now reading as follows “…*Disease for those ART-naïve or returning to care after interruption in treatment) strongly predicts severe morbidity, comorbidities and mortality*”

**Comment 8**

**Methods section**

Check and correct the spacing in,” HIV-infected”, “population-based”, “ART-experienced”. Please do same throughout the document.

**Response**

We appreciate the reviewer’s comment. These have been revised as per the recommendation.

**Comment 9**

The sentence,” KwaZulu-Natal, South Africa has 1.9 million PLWH, 1.1 million of whom are receiving ART (17).” is not clear. Check if it could be written as, “KwaZulu-Natal province in South Africa has 1.9 million PLWH, 1.1 millions of whom are receiving ART in xxxx (year/period) (17).’

**Response**

We thank the reviewer for the suggestion. The sentence has been revised as per the recommendation on line 101-102, now reading as follows “*KwaZulu-Natal province in South Africa has 1.9 million PLWH, 1.1 millions of whom were receiving ART in 2023.*”

**Comment 10**

**Result section** –well narrated yet needs to be condensed/summarized.

Please review this sentence, ”… and Having TB (AOR 2.46; 95% CI 2.19-2.76) was associated with higher odds of AHD at ART initiation (Table 2).”

**Response**

We thank the reviewer for the comment. We have condensed the results section as reflected in lines 170-177, 179-238, and 279-294.

**Comment 11**

Check if heading write up fits to PLOS ONE criteria. E.g 3.1 Discussion, 3. Results ….

**Response**

We appreciate the insightful input and apologize for the oversight. This has been addressed appropriately, as shown in line 332.

**Comment 12**

**Discussion section:-**The interpretation of the finding and the suggestion is well narrated.

Please restructure the discussion paragraph by paragraph (P).

Example: check if this structuring make sense--e.g P1:*17-24*, P2: *25-43* P3:*44-56* P4:*57-67* PY5:*67-73* PY6: *73-79* PY7: *80-88*

Also try to compare and contrast the findings with previous similar studies: For instance, **ART-experience and ADH,**

**Response**

We thank the reviewer for the comment. We have restructured the discussion section paragraph by paragraph and trimmed it as suggested. These changes are reflected in lines 348-369, 378-441, and 448-463.

**Comment 13**

Make similar decimal point description of %. E.g line # 25-30: 36.6%, 62%, 43.45%. These used different decimal points. Suggested to write with same decimal point throughout.

**Response**

We thank the reviewer for the comment. We have addressed these inconsistencies in decimal point reporting as shown in line 373 and 377.

**Comment 14**

**limitation:**

Refer to PLOS ONE journal requirements while narrating this section-which should be condensed/summarized/trimmed

**Response**

We appreciate the reviewer’s comment. We have condensed and summarized this section as recommended, as shown in lines 489-504.

**Comment 15**

Line # 100: you may replace ‘conclusion’ with “interpretation”.

**Response**

We thank the reviewer for this comment, and we have replaced “conclusion” with “interpretation”. This change is shown in line 492-493 as it reads as follows “*However, interpretation should be done with caution due to limitations inherent in observational study designs.”*

**Comment 16**

**Implications for practice and future research**

The content is very important. Yet need to be summarized and be part of discussion above. Again, please refer to PLOS ONE journal structure : [Submission Guidelines | PLOS ONE](https://journals.plos.org/plosone/s/submission-guidelines.#:~:text=The%20platform%20provides%20specialized%20tools%20for%20communicating%20technical%20details,%20including)

**Response**

We appreciate the reviewer’s comment. We have removed implication for practices and future research from the manuscript.
